# Retrotransposition of Long Interspersed Element 1 Induced by Methamphetamine or Cocaine*

**DOI:** 10.1074/jbc.M114.559419

**Published:** 2014-07-22

**Authors:** Noriyuki Okudaira, Yukihito Ishizaka, Hajime Nishio

**Affiliations:** From the ‡ Department of Legal Medicine, Hyogo College of Medicine, 1-1 Mukogawa-cho, Nishinomiya, Hyogo 663-8501, Japan and; § Department of Intractable Diseases, National Center for Global Health and Medicine, 1-21-1 Toyama, Shinjuku-ku, Tokyo 162-8655, Japan

**Keywords:** cAMP Response Element-binding Protein (CREB), Chromatin Regulation, Neurobiology, Pharmacology, Retrovirus

## Abstract

Long interspersed element 1 (L1) is a retroelement constituting ∼17% of the human genome. A single human cell has 80–100 copies of L1 capable of retrotransposition (L1-RTP), ∼10% of which are “hot L1” copies, meaning they are primed for “jumping” within the genome. Recent studies demonstrated induction of L1 activity by drugs of abuse or low molecular weight compounds, but little is known about the underlying mechanism. The aim of this study was to identify the mechanism and effects of methamphetamine (METH) and cocaine on L1-RTP. Our results revealed that METH and cocaine induced L1-RTP in neuronal cell lines. This effect was found to be reverse transcriptase-dependent. However, METH and cocaine did not induce double-strand breaks. RNA interference experiments combined with add-back of siRNA-resistant cDNAs revealed that the induction of L1-RTP by METH or cocaine depends on the activation of cAMP response element-binding protein (CREB). METH or cocaine recruited the L1-encoded open reading frame 1 (ORF1) to chromatin in a CREB-dependent manner. These data suggest that the cellular cascades underlying METH- and cocaine-induced L1-RTP are different from those behind L1-RTP triggered by DNA damage; CREB is involved in drug-induced L1-RTP. L1-RTP caused by drugs of abuse is a novel type of genomic instability, and analysis of this phenomenon might be a novel approach to studying substance-use disorders.

## Teaser

**Background:** Effects of drug abuse on LINE-1 retrotransposition (L1-RTP) in somatic cells are unknown.

**Results:** Methamphetamine and cocaine-induced L1-RTP depends on CREB.

**Conclusion:** Methamphetamine and cocaine activate CREB and L1-RTP in neuronal cell lines.

**Significance:** To our knowledge this is the first study to demonstrate the induction of L1-RTP by drugs of abuse.

## Introduction

Long interspersed element 1 (LINE-1 or L1)^2^ is the most abundant of endogenous retroelements, accounting for ∼17% of the human genome (1, 2). As an autonomous retroelement, L1 can retrotranspose not only itself but also other retroelements such as Alu and short interspersed element variable number tandem repeat *Alu* (SINE-VNTR-*Alu*) (3). A single human cell has >5 × 10^5^ copies of L1, and most of them are functionally defective (4). Nonetheless, 80–100 copies of L1 are capable of retrotransposition (L1-RTP) (4), and ∼10% of these are highly active via the “copy and paste” mechanism termed target site-primed reverse transcription (5). Actual occurrence of L1-RTP activity in the germ line has been estimated to be 1 in 108 births (2, 4), and L1-RTP randomly disrupts genes, leading to congenital mutations (6, 7). Lately, most studies on L1-RTP have focused on early embryogenesis (8–11), and another line of evidence suggests that L1-RTP also occurs in somatic cells (12–15). L1 elements are 6 kb in length and contain 2 open reading frames, 1 and 2 (ORF1 and ORF2) (2, 16), whose molecular masses are ∼40 and 150 kDa, respectively. ORF1 is a basic protein with RNA binding and nucleic acid chaperone activity and is present within cytoplasmic ribonucleoprotein complexes or stress granules in the cytoplasm (17, 18), whereas ORF2 has dual functions of endonuclease (19) and reverse transcriptase (RT) (20). ORF1 and -2 can perform L1-RTP, which proceeds via 3 steps: transcription, reverse transcription, and insertion of the newly synthesized L1 DNA into the host genome via target site-primed reverse transcription (2, 16, 21).

Recent observations suggest that L1-RTP occurs in somatic cells and that copy numbers of L1 are increased in human brain tissues (11, 22). In the mammalian nervous system, L1 can be expressed and can undergo retrotransposition at a high frequency (11, 12, 22). Furthermore, some neurological diseases such as Rett syndrome and ataxia telangiectasia involve deregulation of L1-RTP (22, 23). Because L1-RTP changes cellular properties by causing gene deletions (24, 25), DNA damage (21), apoptosis (26), and immune response (27), deregulation of L1-RTP in somatic cells is likely to function as an etiologic agent of some diseases.

The cAMP response element-binding protein (CREB), as the name suggests, binds to cAMP-responsive element (CRE), which is found in most cAMP-regulated promoters (28–30). CREB activation (*e.g.* Ser^133^ phosphorylation) is mediated by a number of kinases, including phosphorylated extracellular signal-regulated kinase (pERK) (31). CREB is conserved from invertebrates to humans and participates in various physiological functions such as learning, memory, spatial perception, and cellular survival (28–30, 32, 33). CREB regulates the transcription factor-mediated cell signaling cascade, which is involved in the growth process that yields synapse-specific structural changes (34).

Methamphetamine (METH) and cocaine are recreational drugs that cause addiction and are among the most widely used illegal drugs worldwide (35–38). These drugs undergo rapid transport across the blood-brain barrier and as a result have profound effects on the central nervous system (39). The neurotoxicity of METH leads to neuronal cell death in certain brain regions, including the cortex, striatum, and hippocampus (40). METH and cocaine separately increase oxidative stress and production of reactive oxygen species in the monoaminergic systems of the brain (41). As reported by numerous studies, some cell signaling cascades are activated by METH and cocaine, such as the ERK, phosphatidylinositol 3-kinase, V-akt murine thymoma viral oncogene homolog 1 (Akt-1), and CREB pathways. The transcription factor CREB is often induced after chronic abuse of drugs (42–45).

In this study we found that METH and cocaine induce L1-RTP and that the induction of this activity by METH and cocaine depends on CREB. Biochemical analysis revealed that METH or cocaine recruits ORF1 to chromatin. To the best of our knowledge, this is the first study demonstrating the induction of L1-RTP by drugs of abuse. These data support the existence of CREB-mediated genome shuffling via L1-RTP; in this paper we discuss its possible involvement in psychiatric disorders associated with METH and cocaine use.

## EXPERIMENTAL PROCEDURES

### 

#### 

##### Chemicals and Cells

SH-SY5Y (EC94030304), PC12 (EC88022401), HeLa (EC93021013), HT1080 (EC85111505), and SK-N-SH cells (EC86012802) were obtained from DS Pharma Biomedical. SH-SY5Y cells were maintained at 37 °C and 5% CO_2_ in Eagle's minimum essential medium plus Ham's F-12 medium containing 1% nonessential amino acids and 15% fetal bovine serum (final concentration; Hyclone^TM^, Thermo Fisher Scientific). PC12, HeLa, HT1080, and SK-N-SH cells were cultured in Dulbecco's modified Eagle's medium supplemented with 10% fetal bovine serum. The transfection efficiency was ∼70% for HeLa and HT1080 and ∼30% for PC12, SH-SY5Y, and SK-N-SH cells as determined using fluorescence-activated cell sorting on day 2 after transfection of plasmid DNA encoding enhanced green fluorescent protein (EGFP; data not shown). Protease and phosphatase inhibitors were purchased from Roche Diagnostics (Tokyo, Japan). METH and cocaine were purchased from Dainippon Sumitomo Pharma Co., Ltd., and Sanseidou (Japan). A license for METH (no. 25-013) and cocaine (no. 1040) was issued by the governor of Hyogo. We prepared a 100 mm stock solution of the RT inhibitor, stavudine (d4T), in DMSO. About 30 min before the addition of METH or cocaine, d4T was added to a final concentration of 50 μm to the cell culture medium.

Antibodies against H2AX (Millipore, Billerica, MA), γ-H2AX (Millipore), CREB (Cell Signaling Technology Inc., Beverly, MA), phospho-CREB (Ser^133^, Cell Signaling Technology), and glyceraldehyde-3-phosphate dehydrogenase (GAPDH; Trevigen, Gaithersburg, MD) were used as primary antibodies. A rabbit polyclonal antibody against human ORF1 was generated using the peptide MGKKQNRKTGNSKTQSAC as an immunogen (Medical and Biological Laboratories) (46). As secondary antibodies, we used anti (α)-mouse IgG (DAKO Japan, Tokyo, Japan) and α-rabbit IgG (DAKO Japan) antibodies, both of which were conjugated with horseradish peroxidase. For immunohistochemical analysis, Alexa Fluor 555-conjugated goat α-mouse IgG, (Invitrogen) were used as secondary antibodies. Hoechst 33258 was purchased from Invitrogen.

##### L1-RTP Assay

**FIGURE 1.**
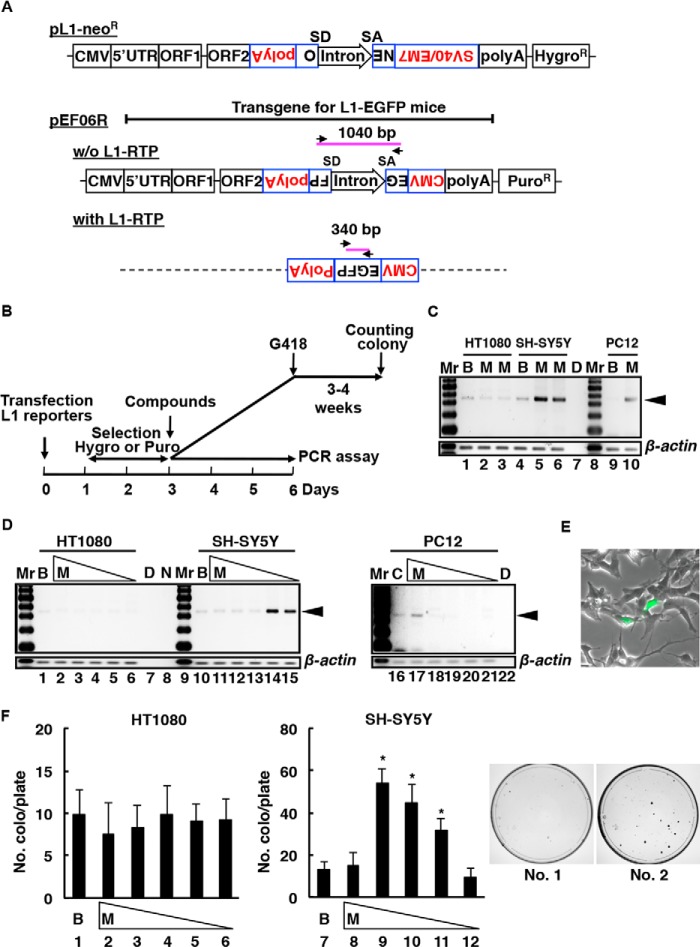
**METH induces L1-RTP.** *A*, schematics of the constructs used for the assay of L1-RTP and the rationale for the PCR-based assay (see details under “Experimental Procedures”). *Arrows* indicate primers for the PCR-based assay. *SD* and *SA* indicate splicing donors and splicing acceptors, respectively. *B*, the experimental protocol for the PCR-based assay and colony formation assay (see details under “Experimental Procedures”). *C*, results of the PCR-based assays. HT-1080, SH-SY5Y, and PC12 cells showed the amplified 340-bp band within 3 days after METH treatment (*arrowheads*). *Mr*, DNA molecular weight markers. *B*, Hepes-buffered saline (HBS) buffer; *M*, METH (*lanes 2* and *5*, 0.13 mm; *lanes 3* and *6*, 0.06 mm; *lane 10*, 0.5 mm; *D*, PCR water control. *D*, the dose response of METH in the PCR-based assays. HT-1080, SH-SY5Y, and PC12 cells showed the 340-bp amplicon 3 days after METH treatment (*arrowheads*). *B*, HBS buffer; *M*, METH (*lanes 2*, *11*, and *17*, 1 mm; *lanes 3*, *12*, and *18*, 0.5 mm; *lanes 4*, *13*, and *19*, 0.25 mm; *lanes 5*, *14*, and *20*, 0.13 mm; *lanes 6*, *15*, and *21*, 0.06 mm); *D*, PCR water control; *N*, a lane without a sample. *E*, METH induced L1-RTP in SH-SY5Y cells. *F*, colony formation assay of METH-induced L1-RTP. Cells were incubated with either HBS buffer (*B*, lane 1) or METH at 0.25, 0.13, 0.06, 0.03, or 0.015 mm (*M*, *lanes 2-*6). At least 2 independent experiments were performed, and representative results are shown. The numbers of colonies are presented as the mean ± S.D. *Asterisks* indicate statistical significance (*p* < 0.01 compared with HBS buffer). Colonies formed after treatment with buffer (*plate No. 1*) or 0.13 mm METH (*plate No. 2*) are also shown.

L1-RTP was assayed as described previously (46–49). For the L1-RTP assays we used two reporter constructs, pEF06R (50) and pCEP4/L1mneoI/ColE1 (pL1-Neo^R^) (24, 51), for semiquantitative or colony formation assays, respectively. Each construct contained all components of human L1 within a single transcriptional unit of either EGFP or Neo^R^, which were inserted in reverse orientation. When L1-RTP occurs, the intron within each reporter gene is spliced out, and then the pEF06R plasmid expresses a functional EGFP, whereas pL1-Neo^R^ expresses a functional neomycin resistance gene (Neo^R^). Cells were transfected with either pEF06R or pL1-Neo^R^ using Lipofectamine 2000 (Invitrogen). The cells were selected for 2 days with puromycin (0.5 μg/ml) for pEF06R or with hygromycin (50 μg/ml) for pL1-Neo^R^. Next the cells were incubated for an additional 2 or 3 days with the indicated amounts of chemicals. For the PCR-based assay, genomic DNA was prepared from harvested cells using a DNA extraction system (Bio-Robot EZ1; Qiagen). For the semiquantitative PCR, primers that were designed to cover exons would amplify a product of ∼1040 bp (without L1-RTP), whereas they would generate a product of ∼340 bp (with L1-RTP) after L1-RTP (Fig. 1*A*). Thus, the occurrence of L1-RTP was determined by evaluating the size of the amplified product. After staining the amplified DNA with SYBR Green I (Lonza, Basel, Switzerland), the signal intensity of the 340-bp bands was measured using a molecular imager (ImageQuant^TM^ LAS 4000 mini; GE Healthcare) and normalized to β-actin (ACTB) bands, used as an internal control. *EFGP* was PCR-amplified using 5′-CTGGTAGTGGTCGGCGAGCTG-3′ and 5′-GACCACCCTGACCTACGGCGT-3′ as the forward and reverse primers, respectively. Amplification reactions were performed using the Ex Taq^TM^ polymerase (TaKaRa) and the following cycling conditions: initial step for 15 min at 94 °C followed by 28–32 cycles of 30 s at 94 °C and 15 s at 68 °C. To detect ACTB as the internal control, 5′-GAGGGAAATCGTGCGTGA-3′ and 5′-AGAAGGAAGGCTGGAAAA-3′ were used as the forward and reverse primers, respectively. PCR amplification was performed using the following cycling conditions: initial 5 min at 94 °C, 20 cycles of 30 s at 94 °C, 30 s at 62 °C, and 1 min at 72 °C. The PCR product was analyzed in agarose gels and visualized with SYBR Green I Nucleic Acid Gel Stain.

In the colony formation assay, ∼2.0 × 10^6^ cells transfected with pL1-Neo^R^ and selected with hygromycin (50 μg/ml) were transferred to 100-mm plates at ∼10^5^ per plate. Next, the cells were incubated for 3 days with the selection and further cultured in the presence of G418 (270 μg/ml). After 3–4 weeks, cell aggregates were stained with methylene blue, and the number of colonies was counted. To determine the occurrence of L1-RTP, each of the six plates was incubated with chemicals or a buffer control, and the number of resulting colonies in each plate was counted.

##### Mutant Plasmids of pL1-Neo^R^

The plasmids listed below contain all components of human L1. The pL1-Neo^R^ vector has a modified mneoI indicator cassette with a bacterial/mammalian promoter and the ColE1 origin of replication (24). The construct JCC9/L1.3-Neo^R^-Asp702Ala (D702A) contains a missense mutation in the reverse transcriptase domain of ORF2. The reverse transcriptase activity of ORF2 is attenuated by the D702A mutation (52). JCC9/L1.3-Neo^R^ was used as an RTP-competent control of the JCC9/L1.3-Neo^R^-D702A construct in a series of experiments.

##### Real-time RT-PCR (qRT-PCR)

qRT-PCR was performed as described previously (46). Total RNA was purified using the RNeasy Mini kit (Qiagen) and treated for 30 min with RNase-free DNase I (Qiagen) at 25 °C. First-strand cDNA was synthesized using random hexamers. The Omniscript^TM^ RT kit (Qiagen) was used for reverse transcription, and amplification of cDNA was monitored and quantified using SYBR Premix Ex Taq (TaKaRa) on an Applied Biosystems 7900HT Fast Real-time PCR system (Invitrogen) according to the manufacturer's instructions. All data were normalized to ACTB. For the quantification of both endogenous and exogenous *ORF2* mRNA, a primer set of L1-EGFP+5653F (5′-CCAAATGTCCAACAATGATAGACTG-3′) and L1-EGFP+5762R (5′-CCATGTCCCTACAAAGGATATGAAC-3′) was used. For the quantification of mature or precursor mRNA of *EGFP*, primer sets of L1-EGFP+6342F (5′- TAGTGGTTGTCGGGCAGCAG-3′) plus L1-EGFP+7351R (5′-TTCAAGATCCGCCACAACATC-3′) or L1-EGFP+7222F (5′-TGGAAGCTGGGTGTGTAGTTATCTG-3′) plus L1-EGFP+7365R (5′-GGCATCAAGGTGAACTTCAAGATC-3′) were used, respectively. For the amplification of ACTB-derived DNA, a primer set of 5′-GAGGGAAATCGTGCGTGA-3′ plus 5′-AGAAGGAAGGCTGGAAAA-3′ was used. To evaluate the levels of mRNA, each sample was analyzed in triplicate, and statistical analysis was performed based on the relative intensity of the mRNA levels normalized to ACTB levels.

**FIGURE 2.**
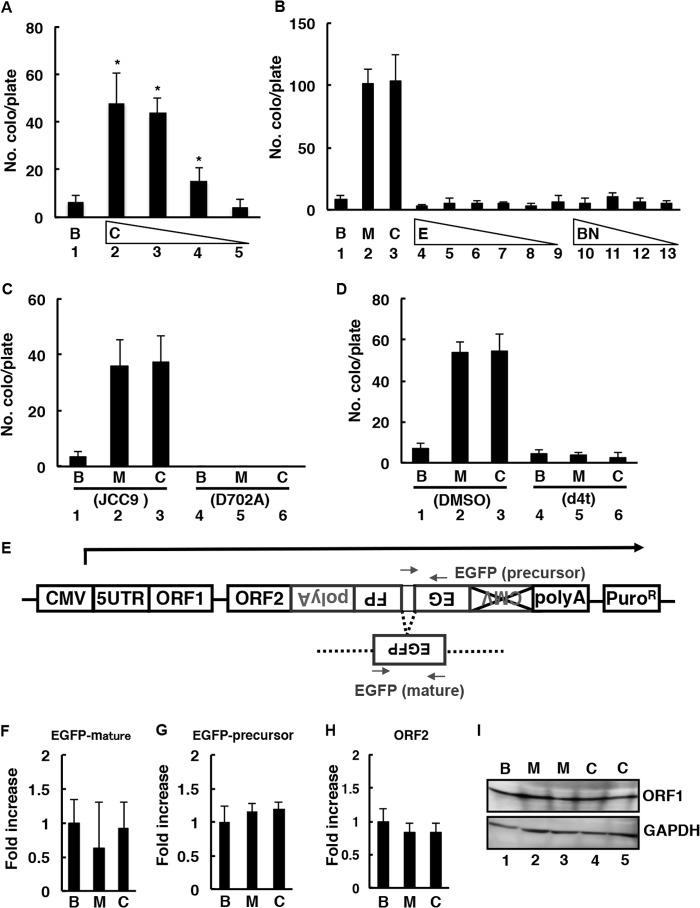
**Cocaine induces L1-RTP, but ethanol and sodium barbital do not.** *A*, colony formation assay of cocaine-induced L1-RTP. Cells were treated with either HBS buffer (*B*, *lane 1*) or cocaine at 0.5, 0.25, 0.13, or 0.06 mm (*C*, *lanes 2–5*). The mean numbers of colonies ± S.D. are shown. *Asterisks* indicate statistical significance (*p* < 0.01 compared with HBS buffer). *B*, colony formation assay showing that ethanol and sodium barbital did not induce L1-RTP. The cells were treated with HBS buffer (*B*, *lane 1*); METH at 0.13 mm (*M*, *lane 2*); cocaine at 0.25 mm (*C*, *lane 3*); ethanol at 1, 0.5, 0.25, 0.13, 0.06, or 0.03% (*E*, *lanes 4-*9); sodium barbital at 2, 1, 0.5, or 0.25 mm (*BN*, *lanes 10–13*). *C*, RT dependence of L1-RTP induced by METH and cocaine. The SH-SY5Y cells had been transfected with pL1-neo^R^ (JCC9 L1 wild type, *lanes 1–3*) or pL1-neo^R^ (JCC9 L1 D702A, *lanes 4–6*). The cells were treated with HBS buffer (*B*, *lanes 1* and *4*), METH at 0.13 mm (*M*, *lanes 2* and *5*), or cocaine at 0.25 mm (*C*, *lanes 3* and *6*). *D*, METH- or cocaine-induced L1-RTP is inhibited by d4T. The cells were treated with DMSO (*lanes 1–3*), d4T (*lanes 4–6*), HBS buffer (*B*, *lanes 1* and *4*), METH at 0.13 mm (*M*, *lanes 2* and *5*), or cocaine at 0.25 mm (*C*, *lanes 3* and *6*). *Asterisks* indicate statistical significance (*p* < 0.01: comparison of *lanes 2* and *5* or *lanes 3* and *6*). *E*, PCR primers for quantitative RT-PCR analysis. A schematic diagram of a construct without the 3′ CMV promoter region. *F* and *G*, no effects of METH or cocaine either on the expression of precursor L1 mRNA or on splicing. HBS buffer control (*B*), 0.13 mm METH (*M*), or 0.25 mm cocaine (*C*). *H*, expression of endogenous *ORF2* mRNA was not induced by either METH or cocaine. *I*, neither METH nor cocaine increased the level of ORF1 expression. GAPDH was included as a loading control. HBS buffer control (*B*, *lane 1*), 0.13 or 0.06 mm METH (*M*, *lanes 2* and *3*), and 0.5 or 0.25 mm cocaine (*C*, *lanes 4* and *5*) are shown. At least two independent experiments were performed, and representative results are shown.

To correctly assess the mature form of L1 mRNA in the case of pEF06R, it was necessary to exclude the effects of the antisense mRNA transcribed from the inverted 3′ CMV promoter (46). For this purpose we prepared a construct that lacked the corresponding region (pEF06R▵3′CMV, see Fig. 2*E*). SH-SY5Y cells were transfected with pEF06R▵3′CMV, and qRT-PCR was performed. The results of 24-h incubation with either METH or cocaine were analyzed.

##### Western Blotting (WB) Analysis

The cells were washed with phosphate-buffered saline (PBS) and resuspended in 50 mm Tris-HCl (pH 7.6), 150 mm NaCl, 1 mm EDTA, 0.1% SDS, 0.5% deoxycholic acid, 1% Nonidet P-40 (radioimmune precipitation assay buffer), and protease inhibitors. After ultrasonication using Bioruptor (UCD-250, Cosmo Bio) for 12.5 min (10 s on, 20 s off) at the middle level of output (250 W) at 4 °C, the soluble cellular extracts were recovered after centrifugation for 10 min at 16,000 × *g*. The protein concentration of each sample was determined using the BCA Protein Assay Reagent kit (Thermo Fisher Scientific), and cell extracts were subjected to WB analysis. The blots were probed with the primary antibody followed by a horseradish peroxidase-conjugated secondary antibody. The immune complexes were visualized using Pierce^TM^ Western blotting Substrate Plus (Thermo Fisher Scientific). WB results were documented and quantified using ImageQuant LAS 4000 mini and Amersham Biosciences Imager 600 (GE Healthcare).

##### ORF1 Proteins Analysis

SH-SY5Y cells were transfected with pEF06R, and either METH or cocaine was added to the culture medium on day 2. Total cell extracts were prepared 24 h after the addition of 0.13 or 0.06 mm METH or 0.5 mm or 0.25 mm cocaine. The extracts were subjected to WB analysis with α-ORF1 antibody as the primary antibody.

##### Assessment of the Effects of Down-regulation of Endogenous Proteins on Induction of L1-RTP

Two small interfering RNAs (siRNAs) were prepared (Invitrogen), and their functions were evaluated by transfection into cells followed by WB analysis. We transfected 10 nm siRNA using Lipofectamine RNAiMAX in SH-SY5Y and SK-N-SH. The nucleotide sequences of each siRNA are shown in Table 1. To evaluate the inhibitory effects of the siRNAs on L1-RTP induction, each siRNA was introduced on day 3 after initial transfection with pL1-Neo^R^. Two days later, the cells were replated, incubated for 2 days with METH or cocaine, and subjected to analysis. As a control, Silencer Negative Control siRNAs (Invitrogen, catalog no. AM4613) were used.

##### Add-back Experiments Using siRNA-resistant cDNA Clones

The basic protocol for these experiments is illustrated in Fig. 1*B*. To evaluate the roles of the target gene products, cells were transfected with CREB siRNA using Lipofectamine RNAiMAX for each gene on day 3 after the first transfection of pL1-Neo^R^. On day 4, pSi^R^-*CREB* (46) was introduced using Lipofectamine 2000, and the cells were again replated on day 5. The cells were then treated with either 0.13 mm METH or 0.25 mm cocaine for 2 days, and G418 selection was started on day 7 after the first transfection with pL1-Neo^R^.

##### Chromatin Recruitment of ORF1

We used the pORF1-TAP (tandem affinity purification) construct (46), which encodes a chimeric protein of ORF1, protein A, and calmodulin-binding protein. On day 2 after transfection of pORF1-TAP into SH-SY5Y cells, either 0.13 mm METH or 0.25 mm cocaine was added to the culture medium, and cell extracts were prepared the following day. The chromatin-enriched fraction (chromatin fraction) was isolated using the Subcellular Protein Fractionation kit (Thermo Fisher Scientific) with micrococcal nuclease as described previously (46, 48, 49). Detection of ORF1-TAP was performed using a horseradish peroxidase-conjugated α-rabbit IgG antibody. H2AX served as an internal control for the chromatin fraction. GAPDH was also assayed to examine the cytoplasmic localization of the proteins.

##### Immunostaining of SH-SY5Y Cells

SH-SY5Y cells were washed with PBS and fixed with 4% paraformaldehyde in PBS. The fixed cells were permeabilized with 0.5% Triton X-100 in PBS for 5 min. After incubation with PBS and 5% bovine serum albumin for 30 min, the cells were incubated with α-γ-H2AX antibodies. After 1 h of incubation at 37 °C, secondary antibodies conjugated with Alexa Fluor 555 (Invitrogen) were added for 1 h at 37 °C. Nuclei were stained with Hoechst 33258 (Invitrogen). The slides were mounted in an anti-fade solution and examined under a fluorescence microscope (BZ-9000; Keyence).

##### Statistical Analysis

Statistical significance was evaluated using the Mann-Whitney *U* test. A *p* value of <0.05 was assumed to mean statistical significance.

## RESULTS

### 

#### 

##### METH and Cocaine Induce L1-RTP Activity

We initially performed PCR-based and colony formation assays using the filtered drug of abuse and the plasmids pEF06R and pCEP4/L1mneoI/ColE1 (pL1-Neo^R^; Fig. 1, *A* and *B*) (24, 50, 51). The induction of L1-RTP activity by METH was also confirmed by a PCR-based assay using pEF06R (46–49) in which the signal intensity of the 340-bp band amplified from a functional EGFP, a product of L1-RTP, was increased by treatment with METH in a human neuroblast cell line (SH-SY5Y cells; Fig. 1*C*, *lane 5*, 0.13 mm, and *lane 6*, 0.06 mm) and a rat pheochromocytoma cell line (PC12 cells; Fig. 1*C*, *lane 10*, 0.5 mm). As shown in Fig. 1*C*, METH induced L1-RTP in different cell lines, including SH-SY5Y and PC12, but did not induce L1-RTP in a human fibrosarcoma cell line (HT1080 cells; Fig. 1*C*, *lane 2*, 0.13 mm, and *lane 3*, 0.06 mm).

No cytotoxic effects of METH at concentrations up to 1 mm were detected (data not shown). We then tested if high doses of METH could induce L1-RTP in HT1080 and SH-SY5Y cells and whether low doses of METH could induce L1-RTP in PC12 cells (Fig. 1*D*). When METH induced L1-RTP, SH-SY5Y cells expressed EGFP (Fig. 1*E*). Next, we evaluated METH-induced L1-RTP using a colony formation assay in which L1-RTP activated the expression of a functional neomycin resistance gene and supported the growth of cells cultured in the presence of the cytotoxic drug, neomycin. According to the experimental procedures shown in Fig. 1*F*, HT1080 and SH-SY5Y cells were transfected with pCEP4/L1mneol/ColE1 (pL1-Neo^R^, Fig. 1*A*), selected on hygromycin, and then treated with METH on day 3 after the transfection. Approximately 10^5^ cells were incubated initially with 0.25–0.015 mm METH. After neomycin selection, we observed 50–100 colonies in each plate (Figs. 1*F* and Fig. 2*B*), indicating that the frequency of L1-RTP induced by METH was ∼1 per 1.0–2.0 × 10^3^ SH-SY5Y cells (*p* < 0.01; Fig. 1*F*, *right panel*). METH induced L1-RTP at a low concentration (0.13–0.03 mm; Fig. 1*F*, *lanes 9–11*). In contrast, METH did not induce L1-RTP in nonneuronal HT1080 and HeLa cells (Fig. 1*F*, *lanes 1–6*, and data not shown).

We next tested whether other drugs of abuse induced L1-RTP, such as cocaine, ethanol, and sodium barbital. We evaluated cocaine-induced L1-RTP at 0.13–0.5 mm using the colony formation assay in SH-SY5Y cells (Fig. 2*A*). After neomycin selection, we observed 50–100 colonies on each plate (Fig. 2, *A* and *B*), indicating that the frequency of L1-RTP induced by cocaine was ∼1 per 1.0–2.0 × 10^3^ in SH-SY5Y cells. On the other hand, ethanol and sodium barbital did not induce L1-RTP in SH-SY5Y cells (Fig. 2*B*). No cytotoxic effects of cocaine, ethanol, or sodium barbital were observed at the concentrations tested (data not shown). Next, using a mutant vector, we tested whether colonies induced by METH and cocaine were dependent on L1-RTP or if these colonies formed nonspecifically. The mutant pL1-Neo^R^ vector used in this experiment was reverse transcriptase-negative (due to a D702A mutation). The results revealed that the colonies did not form at all (Fig. 2*C*). We conducted another experiment using an RT inhibitor (d4T). Previous studies reported a suppressive effect of d4T on L1-RTP (53, 54). The experiment showed that the induction of L1-RTP by METH and cocaine was inhibited by d4T (Fig. 2*D*, *lanes 5* and *6*). Thus, the results suggested that L1-RTP induction by METH and cocaine is a phenomenon dependent on RT.

Moreover, we ruled out the possibility that METH and cocaine induce L1-RTP by up-regulating the expression level of L1 mRNA. qRT-PCR analysis revealed that neither METH nor cocaine increased the splicing efficiency of the immature L1 transcript (Fig. 2, *E*, *F*, and *G*) or *ORF2* mRNA (Fig. 2*H*) or the expression levels of the ORF1 protein (Fig. 2*I*).

##### METH and Cocaine Did Not Induce Double-strand Breaks (DSBs)

**FIGURE 3.**
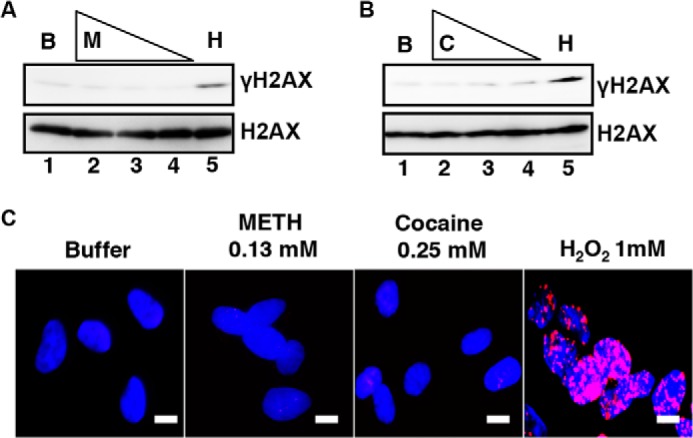
**DNA DSBs are not induced by either METH or cocaine.** *A* and *B*, neither METH nor cocaine induced the expression of γ-H2AX. WB analysis was performed on cells that had been treated for 1 day with 0.13 or 0.06 mm METH and 0.5 or 0.25 mm cocaine. H_2_O_2_ (1 mm, 30 min) was used as the positive control. H2AX was the loading control. HBS buffer control (*B*, *lane 1*); 0.25, 0.13, or 0.06 mm METH (*A*: *M*, *lanes 2–4*) or 0.5, 0.25, or 0.13 mm cocaine (*B*: *C*, *lanes 2–4*); 1 mm H_2_O_2_ (*B*: *H*, *lane 5*). *C*, immunohistochemical assay for cellular markers of DSBs. The results of incubation of SH-SY5Y cells for 1 day with 0.13 mm METH or 0.25 mm cocaine are shown. H_2_O_2_ (1 mm) was used as the positive control. The expression of γ-H2AX phosphorylated at serine 139 was examined. The *scale bar* represents 10 μm.

Farkash *et al.* (50) reported that DNA DSBs caused by γ-radiation stimulate L1-RTP. We tested whether METH or cocaine induced DSBs. SH-SY5Y cells were incubated with 0.25, 0.13, or 0.06 mm METH or cocaine, and then the expression of γ-H2AX was assessed by WB analysis. Neither compound induced the expression of γ-H2AX (Fig. 3, *A* and *B*), and we detected no focus formation of γ-H2AX after treatment of cells with METH at 0.13 mm or cocaine at 0.25 mm (Fig. 3*C*). These observations indicated that induction of L1-RTP by METH and cocaine was attributable to the nongenotoxic effects of these compounds.

##### METH- and Cocaine-induced L1-RTP Is Dependent on CREB

**FIGURE 4.**
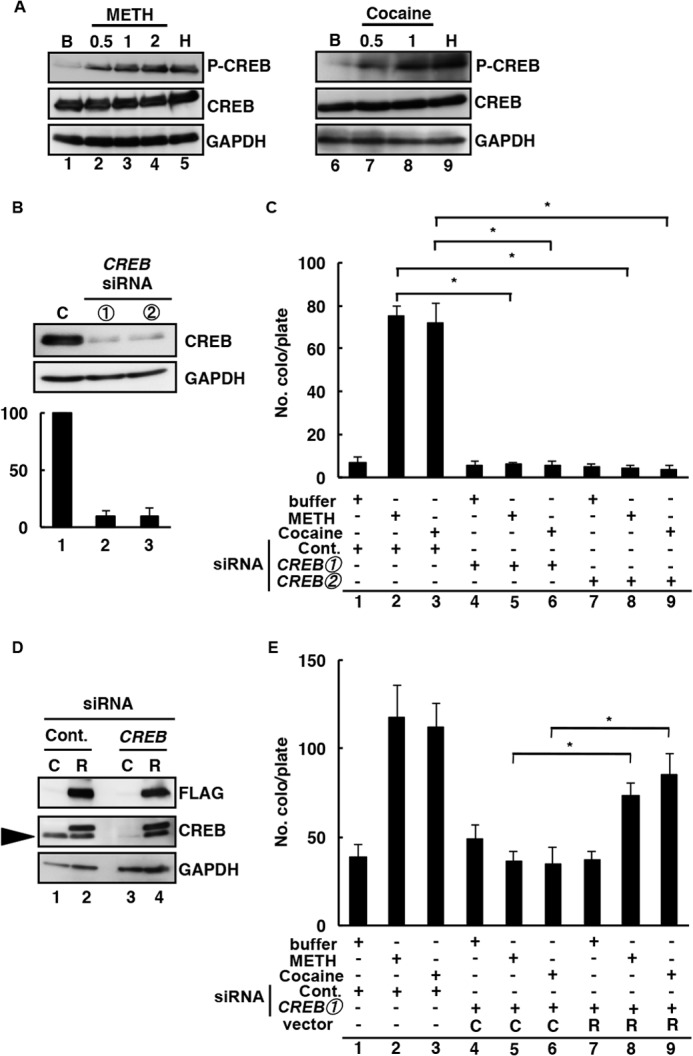
**METH- and cocaine-induced L1-RTP was dependent on CREB protein activity.** *A*, METH or cocaine induced the phosphorylation of CREB. SH-SY5Y cells were incubated with 0.13 mm METH or 0.25 mm cocaine. HBS buffer control (*B*, *lanes 1* and *6*), 0.5, 1, and 2 h of the timeline of 0.13 mm METH (*lanes 2–4*), 0.5 and 1 h of the timeline of 0.25 mm cocaine (*lanes 7* and *8*), and H_2_O_2_ (1 mm) is a positive control (*H*, *lanes 5* and *9*). *B*, effects of *CREB* siRNA on the expression of the endogenous CREB protein. The latter was examined in SH-SY5Y cells that had been transfected with pL1-Neo^R^ and *CREB* siRNA. *Lane 1*, control siRNA (*C*); *lanes 2* and *3*, CREB siRNA-1 or -2. *C*, CREB was required for METH- and cocaine-induced L1-RTP. A colony formation assay was performed for SH-SY5Y cells after the introduction of either control siRNA (*lanes 1–3*) or *CREB* siRNA-1 or -2 (*lanes 4–6* and *7–9*, respectively). The cells were treated with HBS buffer (*lanes 1*, *4*, and *7*), 0.13 mm METH (*lanes 2*, *5*, and *8*), or 0.25 mm cocaine (*lanes 3*, *6*, and *9*). Mean numbers of colonies ± S.D. are shown. The effects of *CREB* siRNAs were significant (*, *p* < 0.02). *D*, pSi^R^-*CREB* restored CREB protein expression. SH-SY5Y cells were transfected with either control siRNA (*lanes 1* and *2*) or *CREB* siRNA-1 (*lanes 3* and *4*) followed by transfection of either a control vector or pSi^R^-*CREB* (*R*). The *arrowhead* represents endogenous CREB, and the upper band is pSi^R^-CREB. *C*, control vector; *R*, pSi^R^-*CREB. E*, pSi^R^-*CREB* restored L1-RTP suppressed by siRNA. SH-SY5Y cells were transfected with *CREB* siRNA followed by the introduction of either a control vector (*lanes 4–6*) or pSi^R^-*CREB* (*lanes 7–9*) the next day. Mean numbers of colonies ± S.D. are shown. The difference between the numbers of Neo^R^ colonies obtained in the pSi^R^-*CREB* experiment and the experiment with the control vector was significant (*, *p* < 0.01: comparison of *lanes 5* and *8* or *lanes 6* and *9*). *C*, control vector; *R*, pSi^R^-*CREB*. At least two independent experiments were performed, and representative results are shown.

To determine the mechanism behind METH- and cocaine-induced L1-RTP, we assessed the involvement of CREB. According to a recent report, METH and cocaine induce the phosphorylation of CREB (55). To confirm this, we performed WB analysis, which showed that both compounds markedly induced the phosphorylation of CREB (Fig. 4*A*). Next we examined the involvement of CREB and found that two *CREB* siRNAs (Table 1; *CREB-1* and *-2*) efficiently suppressed the expression of endogenous CREB (Fig. 4*B*: data with siRNA-1 and -2 are shown) and that both siRNAs abolished METH- or cocaine-induced L1-RTP (Fig. 4*C*, *lanes 5* and *6* and *lanes 8* and *9*). These data suggested that the siRNA abrogated the activity of endogenous CREB and inhibited L1-RTP. To confirm this hypothesis, we carried out an add-back experiment using siRNA-resistant *CREB* cDNA. We confirmed that the transfection of a plasmid DNA expressing a siRNA-resistant FLAG-tagged *CREB* mRNA (pSi^R^-*CREB*) could restore protein expression that had been reduced by the siRNA (Fig. 4*D*, *lane 4*). Subsequently, we found that pSiR-*CREB* restored the formation of Neo^R^ colonies, which had been suppressed by the siRNA (Fig. 4*E*, compare *lanes 5* and *8* and compare *lanes 6* and *9*). Moreover, we confirmed these experiments on another cell line. When the experiments with *CREB* siRNA and added-back siRNA-resistant *CREB* were performed on the SK-N-SH human neuroblast cell line, the results were the same as with SH-SY5Y cells (data not shown). These data indicated that CREB was involved in METH- or cocaine-induced L1-RTP.

**TABLE 1:** **Summary of siRNAs used in the current study** Nucleotide sequences of both sense (Se) and antisense (AS) strands are shown.

Genes		Nucleotide sequences (5′ →3′)
*CREB-1*	Se	GCUGGCUAACAAUGGUACCTT
	AS	GGUACCAUUGUUAGCCAGCTG
*CREB-2*	Se	CCAAUCCCUUGAGUUAUAUTT
	AS	AUAUAACUCAAGGGAUUGGTT

##### Chromatin Recruitment of ORF1 via METH- and Cocaine-dependent Activation of CREB

**FIGURE 5.**
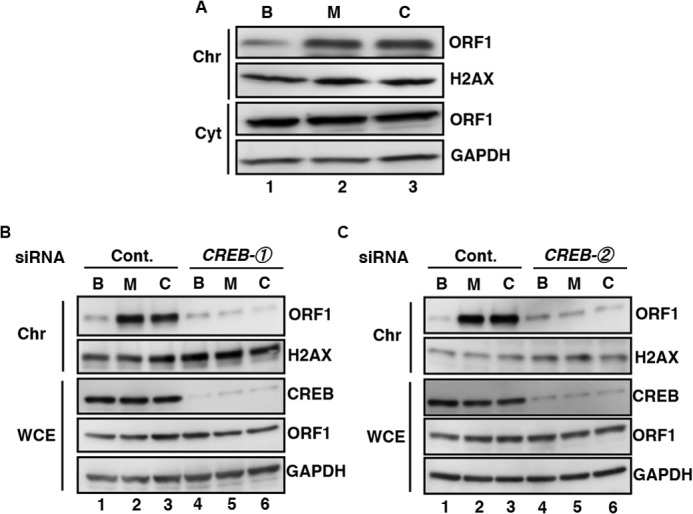
**METH- and cocaine-induced recruitment of L1-ORF1 to chromatin depended on CREB.** *A*, chromatin recruitment of ORF1 by METH or cocaine. After 24 h of incubation with HBS buffer (*lane 1*), 0.13 mm METH (*lane 2*), or 0.25 mm cocaine (*lane 3*), cytoplasmic (*Cyt*) and chromatin (*Chr*) fractions were prepared and analyzed (see “Experimental Procedures”). *B* and *C*, effects of *CREB* siRNA on METH- and cocaine-induced chromatin recruitment of ORF1. SH-SY5Y cells transfected with pORF1-TAP after transfection with either control (*lanes 1–3*) or *CREB* siRNA (*lanes 4–6*). *Lanes 1* and *4*, HBS buffer (*B*); *lanes 2* and 5, 0.13 mm METH (*M*); *lanes 3* and *6*, 0.25 mm cocaine (*C*). Whole cell lysate (*WCE*) and chromatin (*Chr*) fractions were prepared and analyzed. One representative result of two independent experiments performed with 2 types of siRNA is shown.

Goodier *et al.* (18) reported that ORF1, originally located in cytoplasmic stress granules, is translocated to the chromatin in response to stress stimuli. This finding led us to hypothesize that METH or cocaine induces the nuclear trafficking of ORF1. To demonstrate this phenomenon, we performed a subcellular fractionation analysis of ORF1 after transfection of expression vectors encoding a chimeric ORF1-TAP protein. When the transfectants were treated with METH or cocaine, the amount of ORF1 in the chromatin-rich fraction increased without apparent changes in the total amount of ORF1 (Fig. 5*A*, *lanes 2* and *3*). The METH- or cocaine-induced chromatin recruitment of ORF1 was blocked by both types of *CREB* siRNA (Fig. 5, *B*, *lanes 5* and *6*, and *C*, *lanes 5* and *6*). These data indicated that METH and cocaine separately mobilized the L1 component to chromatin via a mechanism involving the activation of CREB.

## DISCUSSION

Recently, the incidence of psychiatric disorders associated with abuse of drugs such as alcohol and METH has been increasing worldwide (35). It is not known what exactly happens at the genomic or chromatin level during this type of abuse and if psychiatric disorders can be caused by these genetic changes. Recent studies on drug abuse employed genome analysis technology and showed that cocaine causes structural changes in chromatin and increases the expression of L1-RTP-associated repetitive elements (56). On the other hand, the cell signaling pathway involved in drug abuse-induced L1-RTP is unknown, and the mechanisms of L1-RTP induction in psychiatric disorders remain elusive. To the best of our knowledge this is the first study demonstrating the induction of L1-RTP by drugs of abuse. Our present work suggests that abuse of drugs functions as a trigger; METH and cocaine reproducibly induce L1-RTP *in vitro*. In this study the induction of L1-RTP was found to be induced by METH or cocaine in neuronal cell lines such as SH-SY5, PC12, and SK-N-SH but not in nonneuronal cell lines such as HeLa and HT1080. Therefore, it appears that METH or cocaine induce L1-RTP only in neuronal cells. METH induced L1-RTP at low concentrations (0.03–0.13 mm; Fig. 1*F*, *lanes 9–11*) and caused fluctuations in the levels of intracellular calcium. When the intracellular calcium concentration increases, calcium interacts with intracellular calcium-binding proteins and produces various physiological effects. Hondebrink *et al.* (57) showed that low concentrations of METH (0.01 mm) stimulate an acetylcholine-evoked calcium increase. Moreover, depolarization-evoked calcium increase is inhibited after exposure to high METH concentrations (1 mm) (57). The change in intracellular calcium concentration influences the expression of some genes (57). Thus, METH at low concentrations activates intracellular signaling and induces L1-RTP. Induction of L1-RTP by METH and cocaine is a phenomenon dependent on RT (Fig. 2, *C* and *D*).

We found that METH- or cocaine-induced L1-RTP are not involved in the mechanisms of DSBs. METH or cocaine did not up-regulate γ-H2AX (H2AX phosphorylated at serine 139) (58), which is a sensitive cellular marker of DSBs. Our results expand the knowledge base in the field of genome instability caused by low molecular weight compounds in relation to L1-RTP activity from tryptophan photoproducts (46) to environmental carcinogens (59).

In a variety of species and types of memory, the process of consolidation of a new memory requires gene expression that begins during training. The proteins of the CREB transcription factor family are evolutionarily conserved regulators of this gene expression (33, 34). CREB is also the major transcription factor induced after chronic abuse of drugs (42, 60). Using an L1-RTP assay, we established that siRNA-mediated suppression of endogenous CREB expression attenuates the induction of L1-RTP activity by METH or cocaine. METH or cocaine promotes the recruitment of ORF1 to chromatin in a CREB-dependent manner. Although activation of CREB is pivotal for METH- or cocaine-induced L1-RTP, our previous study suggested that a simple overexpression of a constitutively active form of *CREB* cDNA does not induce L1-RTP (46). CREB, as a central molecule of memory consolidation, functions in the cAMP/MAPK/CREB transcriptional pathway. Taken together these data suggest that METH and cocaine separately activate CREB and the intracellular cascade of CREB for the induction of L1-RTP. In our present study we were not able to identify the specific factor responsible for the activation of CREB and for the recruitment of ORF1 to chromatin. Moreover, qRT-PCR analysis revealed that METH and cocaine did not increase the expression of L1 mRNA and ORF1 protein (Fig. 2, *F–I*). These findings suggest that CREB functions as the mediator for METH- and cocaine-induced L1-RTP. It is possible that this specific factor induces changes in chromatin structure and relevant transcriptional network, thereby leading to psychiatric disorders.

Recent evidence demonstrated that neurons actually support high levels of L1-RTP (8); for example, L1 expression in neuronal stem cells increases after an environmental change (11, 22), influencing L1-RTP in the hippocampus (61). Bundo *et al.* (62) reported a high L1 copy number, and their experiments with whole-genome sequencing revealed that brain-specific L1 insertion is found in synapses in schizophrenia. The above reports and the results of the present study suggest that abuse of drugs and an environment conduciveness to depression strongly induce L1-RTP in the brain and may be involved in the development of psychiatric disorders. New L1 genomic integrated elements in the neuron could be a part of the mechanism via which METH or cocaine exposure in real life produces a biological effect at a later time point during development, as is the case in several neurodevelopmental syndromes and mental disorders. Some of effects of L1-RTP in neuronal cells are irreversible, and a new insertion may be transmitted to the next generation. The observation that genome modifications induced by L1-RTP specifically happen in neurons adds to the list of factors that affect the plasticity of the central nervous system. Taken together, our data suggest that the CREB transcriptional pathway and its neuronal network might be disturbed after chronic abuse of drugs, which causes L1-RTP and thus affects the plasticity of neurons and promotes the development of substance-use disorders.

## Footnotes

2 The abbreviations used are:

LINE-1 or L1: long interspersed element 1
L1-RTP: LINE-1 retrotransposition
RT: reverse transcriptase
CREB: cAMP response element-binding protein
EGFP: enhanced green fluorescent protein
qRT-PCR: quantitative real-time-PCR
WB: Western blotting
DSB: double-strand break
HBS: Hepes-buffered saline
METH: methamphetamine.
